# Potential Application of Yokukansan as a Remedy for Parkinson's Disease

**DOI:** 10.1155/2018/1875928

**Published:** 2018-12-20

**Authors:** Jung-Hee Jang, Kyungsook Jung, Joong-Sun Kim, Inchul Jung, Horyong Yoo, Changjong Moon

**Affiliations:** ^1^Department of Korean Internal Medicine, Dunsan Korean Medical Hospital, Daejeon University, Daejeon 35235, Republic of Korea; ^2^Immunoregulatory Materials Research Center, Korea Research Institute of Bioscience and Biotechnology, 181 Ipsin-gil, Jeongeup-si, Jeonbuk 56212, Republic of Korea; ^3^K-herb Research Center, Korea Institute of Oriental Medicine, Daejeon 34054, Republic of Korea; ^4^Department of Korean Neuropsychology, Dunsan Korean Medicine Hospital, Daejeon University, Daejeon 35235, Republic of Korea; ^5^College of Veterinary Medicine and BK21 Plus Project Team, Chonnam National University, Gwangju 61186, Republic of Korea

## Abstract

Parkinson's disease (PD), the second most common progressive neurodegenerative disorder, is characterized by complex motor and nonmotor symptoms. The clinical diagnosis of PD is defined by bradykinesia and other cardinal motor features, although several nonmotor symptoms are also related to disability, an impaired quality of life, and shortened life expectancy. Levodopa, which is used as a standard pharmacotherapy for PD, has limitations including a short half-life, fluctuations in efficacy, and dyskinesias with long-term use. There have been efforts to develop complementary and alternative therapies for incurable PD. Yokukansan (YKS) is a traditional herbal medicine that is widely used for treating neurosis, insomnia, and night crying in children. The clinical efficacy of YKS for treating behavioral and psychological symptoms, such as delusions, hallucinations, and impaired agitation/aggression subscale and activities of daily living scores, has mainly been investigated in the context of neurological disorders such as PD, Alzheimer's disease, and other psychiatric disorders. Furthermore, YKS has previously been found to improve clinical symptoms, such as sleep disturbances, neuropsychiatric and cognitive impairments, pain, and tardive dyskinesia. Preclinical studies have reported that the broad efficacy of YKS for various symptoms involves its regulation of neurotransmitters including GABA, serotonin, glutamate, and dopamine, as well as the expression of dynamin and glutamate transporters, and changes in glucocorticoid hormones and enzymes such as choline acetyltransferase and acetylcholinesterase. Moreover, YKS has neuroprotective effects at various cellular levels via diverse mechanisms. In this review, we focus on the clinical efficacy and neuropharmacological effects of YKS. We discuss the possible mechanisms underpinning the effects of YKS on neuropathology and suggest that the multiple actions of YKS may be beneficial as a treatment for PD. We highlight the potential that YKS may serve as a complementary and alternative strategy for the treatment of PD.

## 1. Introduction

Parkinson's disease (PD) is a chronic, progressive neurodegenerative disorder characterized by neuronal loss in the substantia nigra resulting in striatal dopamine deficiency [1]. PD is the second most common neurodegenerative disorder and occurs in 2-3% of people older than 65 years [1]. PD is typified by motor symptoms such as tremor, rigidity, bradykinesia, and postural instability. Additionally, most patients with PD experience nonmotor symptoms such as sleep disorders, cognitive impairments, disorders of mood and affect, autonomic dysfunction, sensory symptoms, and pain [1]. Thus, PD requires continued treatment to prevent deterioration of the quality of life.

The gold standard therapy for PD is levodopa (L-DOPA), although the long-term use of L-DOPA and dopamine agonists causes diminished efficacy and side effects such as motor complications, neuropsychiatric symptoms, and sleep disturbances [2]. Therefore, novel therapies without the limitations of the current standard therapy for PD are required to enable better management of patients with neurodegenerative disorders and improve their quality of life. The dopamine system is the main pharmacological target for the treatment of PD, as disease pathogenesis is characterized by a loss of dopaminergic neurons in the substantia nigra pars compacta [1]. Additionally, neurotransmitter systems in PD-related brain regions, such as the glutamatergic, adenosinergic, noradrenergic, serotonergic, GABAergic, opioidergic, cholinergic, and histaminergic systems, are also involved in PD symptoms [3] and may therefore serve as target candidates for PD pharmacotherapy.

Yokukansan (YKS), also referred to as Yi gan san (YGS), is traditionally used to treat insomnia, night crying in children, and neurosis in Japan and China [3]. YKS consists of seven medical herbs, including Atractylodes lancea rhizome (4.0 g, rhizome of* Atractylodes lancea* De Candolle,* Compositae*), Poria sclerotium (4.0 g, sclerotium of* Poria cocos *Wolf,* Polyporaceae*), Cnidium rhizome (3.0 g, rhizome of* Cnidium officinale* Makino,* Umbelliferae*), Uncaria Hook (3.0 g, thorn of* Uncaria rhynchophylla* Miquel,* Rubiaceae*), Japanese Angelica root (3.0 g, root of* Angelica acutiloba* Kitagawa,* Umbelliferae*), Bupleurum root (2.0 g, root of* Bupleurum falcatum* Linné,* Umbelliferae*), and Glycyrrhiza (1.5 g, root and stolon of* Glycyrrhiza uralensis* Fisher,* Leguminosae*) and its methanol fractions have been shown to contain 25 ingredients [4].

YKS has been reported to be clinically effective for the behavioral and psychological symptoms of dementia (BPSD). In particular, it improves NPI subscale measures, such as delusions, hallucinations, and agitation/aggression subscales and activities of daily living scores in patients with BPSD [57]. Furthermore, the therapeutic effects of YKS have been established for sleep disturbances in patients with dementia [5], neuropathic pain [20], and tardive dyskinesia (TD) [21]. Neuropharmacological studies in animal models have improved our understanding of the therapeutic effects of YKS [3]. For instance, YKS has been shown to inhibit neuronal degeneration, increase the expression of glutamate transporters in the cerebral cortex [46], and ameliorate aggression, anxiety, and hallucinations via modulation of the serotonin receptors 5-HT_1A_ and 5-HT_2A_ in the prefrontal cortex [3]. YKS also inhibited TD via inhibition of excessive extracellular glutamate in the rat striatum [51] and prevented dopaminergic neuronal loss in the nigrostriatum of 1-methyl-4-phenyl-1,2,3,6-tetrahydropyridine- (MPTP-) treated mice [58], which is an animal model of PD [59, 60]. Additionally, YKS inhibited cytotoxicity in various* in vitro* models of neurodegeneration [52–55].

Although clinical trials of YKS have been conducted for various diseases, it is believed that YKS has more general effects on neuropsychiatric and sleep disturbance symptoms [2, 5–18]. Here, we review the potential benefits of YKS on PD symptoms because it may be effective for the nonmotor symptoms. Although studies proving the efficacy of YKS for treating PD are limited, we highlight findings from various PD models that have shed light on the possible mechanisms that might underlie the pharmacotherapeutic effects of YKS for PD.

## 2. Clinical Effects of YKS on PD-Like Symptoms

Several clinical studies have identified effects of YKS on PD-like symptoms in various neurological disorders (Table 1).

### 2.1. Sleep Disturbances

Sleep disruption in PD starts early in the disease progression and is caused by multiple factors, such as abnormalities in primary sleep architecture, nocturia, and restless legs syndrome causing arousal. Relevant subcategories of sleep disorders are rapid eye movement (REM) sleep behavior disorder (RBD), represented by an absence of REM atonia, dream-enacting behavior, and excessive daytime sleepiness [61]. In normal healthy adults, Yokukansankachimpihange (YKSCH), which comprises YKS and two additional herbs (compared to Anchu-san), increased total sleep time and sleep efficiency based on polysomnography (PSG) recordings [7]. Additionally, YKS has been reported to be beneficial for sleep disturbance. It ameliorated sleep disorders as assessed by the neuropsychiatric inventory (NPI) and actigraphic evaluations in patients with Alzheimer's disease (AD) [5] and improved sleep quality as assessed via PSG and the Pittsburgh Sleep Quality Index in patients with dementia [6]. YKS also suppressed RBD, which is characterized by parasomnia, an absence of REM atonia, and dream-enacting behavior [8]. Collectively, these findings suggest that YKS may have therapeutic effects on insomnia, which is a nonmotor symptom of PD.

### 2.2. Neuropsychiatric and Cognitive Impairments

The neuropsychiatric and cognitive symptoms of PD include anxiety, depression, hallucinations, and cognitive deficits [61]. In patients with PD or PD with dementia (PDD), administration of YKS for 4 or 12 weeks improved the total NPI score, which evaluates BPSD and subscale hallucinations [2, 9]. The long-term administration of YKS (12 weeks) also improved subscale anxiety and apathy scores [2]. Based on the Mini-Mental State Examination (MMSE), used to assess cognitive function, YKS treatment produced slight improvements in outcomes in patients with PDD, but not in those with PD. Additionally, treatment with YKS did not alter motor function based on the Unified Parkinson Disease Rating Scale-III (UPDRS III) for determining mobility in PD and the Hoehn–Yahr score for evaluating PD severity [2, 9].

In four clinical studies, NPI and Neuropsychiatry Inventory-Nursing Home version total scores were improved and MMSE was not changed in patients with dementia treated with YKS for 4 or 8 weeks [6, 10–12]. Additionally, in four clinical studies of the effects of YKS treatment for 4 or 12 weeks on patients with AD, total NPI scores improved in three [14–16]. Furthermore, NPI Brief Questionnaire Form (NPI-Q) scores, a simpler evaluation tool for BPSD, did not change in a randomized placebo-controlled multicenter trial [13]. Thus, the NPI-Q may be inappropriate for evaluating the effect of YKS treatment in mild BPSD. Additional studies have revealed that the MMSE, Zarit Burden Interview (ZBI), and Self-rating Depression Scale (SDS) scores are not improved by YKS treatment [13–16]. This lack of improvement in ZBI for evaluating the burden of caregivers and the SDS for evaluating caregiver's depression might have been due to the relatively short duration (4 weeks) of YKS administration in the above-mentioned studies [15]. These studies did reveal a difference in the subscale items in each study for patients with PD, dementia, and AD, as well as improvements in total NPI scores and in the scores of the specific NPI subscale that measures neuropsychiatric symptoms [13–16]. However, YKS did not effectively improve cognitive or motor function. In addition, the outcome of the specific evaluation index is dependent on the duration of YKS administration.

In vascular dementia patients, the effect of YKS was similar in patients with PD, dementia, and AD with regard to improvements in NPI, but without changes in MMSE, the Barthel Index for activities of daily living, or the Disability Assessment for Dementia [17]. In very-late-onset schizophrenia-like psychosis, YKS treatment significantly improved all measures of psychotic symptomatology, including the psychiatric rating scale, clinical global impression scale-severity, and positive and negative syndrome scale scores, but did not significantly alter abnormal movements, as determined by the Simpson-Angus scale, Barnes Akathisia scale, and the involuntary movement scale [18]. Consequently, the therapeutic effects of YKS predominantly alter neuropsychiatric symptoms across various neurological disorders and may thus improve BPSD clinically.

Several studies have examined improvements in cognitive function following YKS treatment. In most studies, YKS treatment did not affect MMSE scores (in terms of measurements of cognitive function), while it did improve cognitive function in daily life and per the Brief Assessment of Cognition in Schizophrenia, Japanese Version score in a schizophrenia case report [19]. Additionally, this effect of YKS may be mediated by serotonin (5-HT) transmission and the amelioration of aberrant glutamate transmission [19]. As mentioned above, administration of YKS induced slight improvements in cognitive function in patients with PDD [9]. Future studies should examine the effects of YKS on cognitive function using a variety of evaluation indexes.

### 2.3. Pain

Pain is a common symptom experienced by patients with PD and is associated with motor fluctuations and early morning dystonia [61]. Central neuropathic pain has been described in patients with PD, but has a low incidence. Additionally, while L-DOPA does not exert an analgesic effect on pain [62], YKS has been found to be clinically effective for use in patients with neuropathic pain (significant decreases in the visual analogue scale and pain scores after treatment) [20]. However, further studies are needed to validate the effects of YKS and its underlying mechanism(s) of action in the context of pain.

### 2.4. Tardive Dyskinesia

PD is characterized by bradykinesia and cardinal motor features such as a resting tremor, rigidity, and postural instability [1]. Although the presence of tardive parkinsonism is controversial, drug-induced parkinsonism is not uncommon in patients treated with dopamine receptor-blocking agents [63]. TD is characterized by abnormal, involuntary, irregular choreoathetoid muscle movements in the head, limbs, and trunk. Critically, YKS improved TD in patients with schizophrenia who had neuroleptic-induced TD [21]. Administration of YKS in patients with schizophrenia similarly improved their TD and psychotic symptoms [21].

## 3. Protective Effects of YKS on PD-Like Symptoms in Animal Models

Several preclinical studies have attempted to clarify the effects of YKS on PD-like symptoms using various animal models of neurological disorders (Table 2).

### 3.1. Sleep Disturbances

A previous study using the pentobarbital-induced sleep test and electroencephalogram analysis reported sleep promotion via regulation of GABA_A_ receptors and GABA content with 5-hydroxytryptophan [64]. YKS enhanced pentobarbital-induced sleep in socially isolated mice, which have shorter sleeping times than do group-housed mice. This effect of YKS was reversed by bicuculline (a GABA_A_-receptor antagonist), suggesting that the GABA_A_-benzodiazepine receptor complex is involved in the sleep-promoting effect of YKS [22]. Additionally, a recent study showed that a drop in body temperature was responsible for promoting sleep and that YKS has a sleep-promoting effect via decreases in body temperature based on thermography used to screen sleeping substances [23].

### 3.2. Neuropsychiatric Symptoms

#### 3.2.1. Depression

Depression affects 10-45% of patients with PD and is the most important predictor of quality of life in patients with PD [61]. Chronic stress is a well-known risk factor for depression [24]. Furthermore, brain glutamatergic neurotransmission is involved in the pathogenesis of stress-related depression. The excitatory amino acid transporter (EAAT), which modulates glutamate levels in the synaptic cleft, is decreased in the hippocampus of stress-maladaptive mice, an effect that was ameliorated by YKS. YKS also inhibited decreased expression of EAAT2 in the hippocampus of stress-maladaptive mice, as found using western blot analysis, and improved depressive symptoms [24].

#### 3.2.2. Anxiety

Anxiety is a common symptom in PD that can manifest as panic attacks and phobias [61]. Previous studies have reported an anxiolytic effect of YKS in animal models. In the elevated plus maze (EPM) test, administration of YKS or YKSCH attenuated freezing duration [25] and increased the time spent in the open arm [26, 27, 29, 31], indicating an amelioration of anxiety-like behavior. In the contextual fear conditioning (CFC) test, YKS reduced freezing behavior (an anxiety response) [27, 30]. Based on locomotor activity measurements, YKS improved anxiety-related responses, such as increased defecation [26], reduced rearing behavior in the open field test [28], and reduced time in the dark box in the light/dark test [29].

To elucidate the mechanisms underlying the anxiolytic effects of YKS, several studies have investigated changes in neurotransmitter systems, such as dopamine and serotonin, as well as c-Fos, as a marker neuronal activation expression induced by YKS. Aging is known to increase anxiety, per increased defecation and decreased time spent in the open arm of EPM, as well as altered extracellular concentrations of serotonin and dopamine [26]. Administration of YKS in aged rats increased extracellular concentrations of serotonin and dopamine in the PFC [26]. Several studies have investigated changes in the 5-HT_1A_ and 5-HT_2A_ serotonin receptors following YKS administration [27, 28, 30]. Furthermore, the anxiolytic effects of YKS in the CFC test were antagonized by a 5-HT_1A_ receptor antagonist (WAY-100635) [27], and 5-HT_1A_ receptor density in the PFC of socially isolated mice was significantly increased by YKS [28]. Moreover, YKS had an antagonistic effect on wet-dog shakes induced by a 5-HT_2A_ agonist, 1-(2,5-dimethoxy-4-iodophenyl)-2-aminopropane (DOI) [29]. Additionally, cotreatment with YKS and fluvoxamine (5 mg/kg, i.p.) specifically decreased 5-HT_2A_ receptor expression in the PFC [30]. Therefore, the anxiolytic effects of YKS may be dependent on 5-HT_1A_ receptor signaling and decreased 5-HT_2A_ receptor expression. Additionally, c-Fos expression has been shown in brain circuits related to anxiety, depression, and stress responses. For example, c-Fos expression was increased in the PFC by YKS but reduced in the prelimbic cortex and amygdaloid nuclei [31]. These results suggest that the effects of YKS are associated with attenuated neuronal activity in the PFC and amygdala [31].

#### 3.2.3. Hallucinations

Hallucinations are present in 30-60% of patients with PD, caused by the side effects of treatment for PD and neuronal degeneration of the pedunculopontine nucleus, locus coeruleus, and raphe nuclei [65]. Scarce evidence exists regarding the effect of YKS on hallucination-like symptoms in animal models. Recently, isolation stress was found to enhance a 2,5-dimethoxy-4-iodoamphetamine (DOI; 5-HT2A receptor agonist)-induced head twitch response, which is considered to be a hallucination-like symptom in mice [32]. Furthermore, 5-HT_2A_ receptors seem to be involved in hallucinations based on 5-HT_2A_ receptor-evoked head-twitches in mice [66], an effect that is increased by elevated corticosterone levels during chronic isolation stress [67]. Several behavioral studies have confirmed the involvement of 5-HT_2A_ receptor signaling in hallucination-like symptoms. For example, DOI-induced head twitch response is induced by a 5-HT_2A_ receptor agonist and suppressed by a 5-HT_2A_ receptor antagonist [32]. Moreover, wet-dog shakes and head twitch are both evoked by the administration of 5-HT_2A_ receptor agonists [66, 68, 69]. In isolation-stressed mice, YKS treatment decreased hallucination-like behaviors and 5-HT_2A_ receptor density in the PFC [32, 33]. Therefore, YKS may improve hallucinations, although it is necessary to develop animal models capable of differentiating between the symptoms of hallucination and anxiety to better understand these outcomes.

#### 3.2.4. Aggressive Behavior

Aggression is a behavioral and psychological symptom of both dementia and PD. In various animal models, aggressive behavior is induced by social isolation, injection of amyloid *β* (A*β*), cholinergic degeneration into the nucleus basalis of Meynert (NBM; an area of the substantia innominata of the basal forebrain containing acetylcholine [ACh] and choline acetyltransferase [ChAT]), para-chloroamphetamine (PCA) injections, and a zinc-deficient diet. Aggression is typically assessed using aggression and resident-intruder tests [34–39].

Alterations in dopaminergic and noradrenergic systems have also been implicated in aggression [70]. YKS ameliorated methamphetamine-induced hyperlocomotion mediated by the dopaminergic system (methamphetamine increases extracellular dopamine) [34]. Additionally, the 5-HT_1A_ receptor exhibits agonistic action via YKS [36] and ionotropic glutamate and GABA_A_ receptors are involved in social isolation-induced aggressive behavior [38]. YKS treatment ameliorated aggression via 5-HT_1A_ receptor stimulation [36, 37] and increased glutamate and GABA concentrations in the resident-intruder test [38, 39].

Furthermore, glucocorticoids are known to be involved in the regulation of neurotransmission. Glucocorticoids enhance excitability of glutamatergic neurons and increase cytosolic Ca^2+^ concentrations which is consequently related to excitotoxicity in the hippocampus [71]. Among the constituents of YKS, geissoschizine methyl ether (GM), a component of Uncaria Hook, and 18*β*-glycyrrhetinic acid (GA), a component of glycyrrhizin, ameliorated increases in glutamate release via attenuation of intracellular Ca^2+^ levels increased by KCl [40]. Thus, YKS may ameliorate social isolation-induced aggressive behavior by attenuating glucocorticoid secretion [39].

#### 3.2.5. Cognitive Impairments

YKS treatment has improved cognitive function in various animal models of diseases such as AD, cerebral ischemia, schizophrenia, aging, and thiamine-deficiency [41–47]. Several studies have examined the potential cognition-enhancing effects of YKS via its effects on the cholinergic system, which plays an important role in cognition [72]. The death of hippocampal pyramidal neurons induced by repeated ischemia (RI) involves downregulated ACh signaling and induces memory impairments [73]. YKS treatment, however, plays a neuroprotective role on the prevention of apoptosis in pyramidal neurons of CA1 and improves memory impairments by increasing ACh levels in the dorsal hippocampus [42].

High [K^+^] concentration and dynamin 1 expression are also implicated in presynaptic vesicular recycling, ChAT activity, and decreased acetylcholinesterase (AChE), enzymes involved in ACh degradation [42]. Elevated [K^+^] evokes the release of stored ACh via increased presynaptic vesicular recycling [74] and ChAT activity [75]. Interestingly, the combination of A*β* oligomers and cerebral ischemia in rats attenuated this response to elevated [K^+^]-evoked ACh release and mimics cognitive impairment in early AD [43]. Thus, YKS treatment may induce elevated [K^+^]-evoked ACh release and thus alleviate some RI-induced memory deficits.

Dynamin 1, a presynaptic protein implicated in early synaptic deficiencies [76], is decreased in a model of cerebral ischemia and was previously associated with memory loss prior to apoptotic neuronal loss in early AD. YKS restored dynamin 1 expression and increased ACh release [43]. ACh levels are also modulated by ChAT or AChE [42]. Olfactory bulbectomy (OBX) in mice causes olfactory loss, increased locomotor activity, aggressiveness, and impaired learning and memory. YKS treatment improved cognitive deficits following degeneration of the cholinergic system induced by OBX [44]. Furthermore, YKS treatment counteracted downregulation of ChAT and muscarinic receptor M_1_ expression in the hippocampus in mice with OBX [44]. Dopaminergic and glutamatergic systems are involved in cognitive impairment [45, 77]. YKS treatment may further ameliorate cognitive impairments by modulating dopaminergic mechanisms, such as reducing the ameliorative effect of YKS by dopamine D1 receptor antagonism, and inhibiting glutamate excitotoxicity, such as inhibiting extracellular glutamate elevations in the ventral posterior medial thalamus in thiamine-deficient rats [45, 46]. Moreover, YKS inhibits inflammatory responses, oxidative damage, and neuronal death via inhibition of microglial activation, oxidative DNA damage, and promotion of neurogenesis in the hippocampal dentate gyrus [47, 48]. Microglial activation and inflammation promote expansion of certain cell populations [78] and may be detrimental to the survival of new hippocampal neurons. Therefore, YKS treatment may ameliorate cognitive deficits via antiapoptotic and anti-inflammatory actions [47, 48].

### 3.3. Pain

Previous studies have attempted to determine the effects of YKS on neuropathic pain. For instance, YKS treatment inhibited mechanical allodynia of a brush in the von Frey filament test [49, 50] and cold allodynia of the acetone test [49] in both a rat model of chronic constriction injury [49] and a mouse model of partial sciatic nerve ligation (PSL), both neuropathic pain models [50]. Glutamatergic neurotransmission and spinal IL-6 expression are known to play important roles in neuropathic pain [49]. Therefore, YKS-induced alleviation of neuropathic pain may be mediated via attenuation of glutamate levels in cerebrospinal fluid dialysate via blockade of glutamate transporters in the rat spinal cord with chronic constriction injury [49] and reduced expression of spinal* IL-6* mRNA in mice with PSL [50].

### 3.4. Tardive Dyskinesia

After injection with haloperidol decanoate for the induction of vacuous chewing movements (VCMs) in long-acting depot neuroleptic-treated rats, YKS ameliorated VCM (a single mouth opening in the vertical plane), which is an index for TD in animal models [51]. Furthermore, YKS treatment inhibited increases in extracellular glutamate concentrations and decreased glutamate transporter (*GLT-1*) mRNA expression in the striatum in haloperidol decanoate-treated rats [51]. However, TD is not a major motor symptom involved in PD [63] and is necessary to validate the effects of YKS in animal models that demonstrate the cardinal motor symptoms of PD.

## 4. In Vitro Neuroprotective Effects of YKS

Previous studies have revealed multiple mechanisms by which the neuroprotective effects of YKS act in various* in vitro *systems (Table 3).

### 4.1. Neuroprotection against Cytotoxicity

Corticosterone (CORT) inhibits cell proliferation and induces cytotoxic effects by modulating transcriptional responsivity. Plasma CORT levels increase in response to stressful conditions and may thus underlie neurological disorders, including neurosis and depression, via stimulation of endogenous stress responses [52]. In a previous study, YKS was demonstrated to inhibit increased aggressive behavior and CORT and orexin levels in rats stressed by individual housing [79]. In an* in vitro* system, YKS was also found to have a neuroprotective effect on CORT-induced cytotoxicity in mouse hippocampal neurons, potentially by ameliorating CORT-induced inhibition of glucose metabolism [52]. In addition, A*β* is known to induce cytotoxicity and serve as a causative molecular mechanism underlying AD [41]. YKS increased cell viability against A*β*-induced cytotoxicity in a primary culture of rat cortical neurons [53, 56]. Therefore, YKS may exert neuroprotective effects on CORT- and A*β*-induced cytotoxicity.

### 4.2. Oxidative Stress

PC12 cells are used to assess oxidative stress in neurodegenerative disorders such as PD, AD, and Huntington's disease [54]. Glutamate-induced toxicity causes oxidative stress by reducing intracellular levels of glutathione (GSH). YKS protected against PC12 cell death evaluated via the 3-(4,5-dimethylthiazol-2-yl)-2,5-diphenyltetrazolium bromide (MTT) assay and ameliorated reductions in intracellular GSH levels, thereby preventing oxidative injury due to glutamate-induced oxidative stress [54]. Furthermore, YKS inhibited neuronal degeneration and increased expression of glutamate transporters in the cerebral cortex [46] and ameliorated aggression, anxiety, and hallucinations via modulation of 5-HT_1A_ and 5-HT_2A_ receptors in the PFC [3].

YGS40, an active fraction of YGS, prevented oxidative stress by decreasing cytotoxicity. This was confirmed using MTT and lactate dehydrogenase (LDH) assays. YGS40 also protected against H_2_O_2_-induced apoptosis in PC12 cells. Hydrogen peroxide (H_2_O_2_), the main component of reactive oxygen species (ROS), can cause oxidative stress and induce apoptosis. YGS40 prevented mitochondrial damage, such as MMP loss, by H_2_O_2_-induced apoptosis. Furthermore, YGS40 protected intracellular enzyme superoxide dismutase activity from antioxidants and decreased levels of malondialdehyde, a marker of lipid peroxidation [55].

### 4.3. Neurotransmission

To overcome the limitations of antipsychotic medicines such as extrapyramidal symptoms and other adverse events, YKS has been used therapeutically for BPSD. The serotonergic system plays an important role in BPSD pathophysiology and is implicated in cognitive dysfunction. Human recombinant 5-HT_1A_ receptors were expressed in the membrane of Chinese hamster ovary (CHO) cells and [3H] 8-OH-DPAT was used as a competitive radioligand to assess 5-HT_1A_ receptor binding. YKS prevented radioligand binding to 5-HT_1A_ receptors and had a partial agonistic effect on 5-HT_1A_ receptors in CHO cells [56]. These results may shed light on the neuropharmacological mechanisms of YKS and further suggest that YKS may be a therapeutic candidate for BPSD.

## 5. Relevance of YKS to Autonomic Dysfunctions

The nonmotor symptoms (i.e., cardiovascular and urinary dysfunctions induced by dysautonomia) of PD have been studied in both patients and animal models [61, 80–82]. The increased prevalence of cardiovascular dysfunction in early stage PD patients has been confirmed with evidence of reduced total power spectral analysis of heart rate at rest and observations of mild degrees of exercise intolerance in these patients [80]. Additionally, urinary dysfunction in PD includes symptoms such as urgency, frequency, nocturia, and urge incontinence [83].

Dysautonomia is an important symptom that is primary complaint of PD patients and significantly impairs their quality of life. However, little is known about the effects of YKS on clinical and preclinical autonomic dysfunction in cardiovascular and urinary systems. Only one previous case study of the effectiveness of YKS in nocturnal enuresis in children has been conducted. Interestingly, in a child with monosymptomatic nocturnal enuresis who did not response to desmopressin, which is the primary therapy for nocturnal enuresis, YKS with desmopressin was shown to be effective [84]. However, this case of pediatric monosymptomatic nocturnal featured no other lower urinary tract symptoms or history of bladder dysfunction or PD-like symptoms. Given this limitation, the effects of YKS on dysautonomias in PD patients require further study.

## 6. Conclusion

PD is characterized by motor symptoms (e.g., tremor, rigidity, bradykinesia, and postural instability), nonmotor symptoms (e.g., sleep disorders, cognitive impairments, disorders of mood and affect, autonomic dysfunction, sensory symptoms, and pain), and drug-induced adverse events. L-DOPA, the gold standard drug for the treatment of PD, has a short half-life, resulting in discontinuous drug delivery quick dissipation of its effects. Furthermore, L-DOPA is known to cause complications such as motor response oscillations and drug-induced dyskinesia [1]. Moreover, antipsychotic drugs for BPSD often induce extrapyramidal symptoms and increased mortality among elderly patients [53, 56]. The development of complementary alternative therapies may thus help to mitigate the symptoms of PD and circumvent the need to increase standard medication doses as well as minimize any adverse events related to conventional medication use.

**Figure 1:**
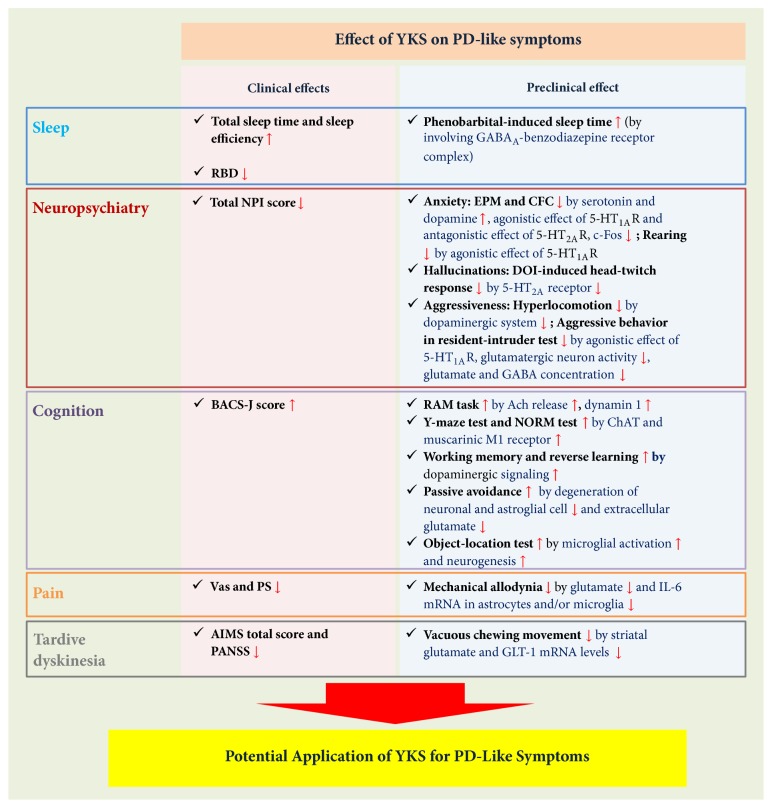
Schematic flow diagram of the effects of YKS on PD-like symptoms. Clinical and preclinical effects of YKS on symptoms such as sleep disturbance, neuropsychiatry, cognition impairment, pain, and tardive dyskinesia is reported. Multiple mechanisms by which YKS exerts neuroprotective effects identified via regulation of neurotransmission and suppression of neuroinflammation. YKS has a potential application for therapy for neurodegenerative diseases such as PD. RBD=rapid eye movement (REM) sleep behavior disorder; NPI=neuropsychiatric inventory; EPM=elevated plus maze; CFC=contextual fear conditioning; DOI=2,5-dimethoxy-4-iodoamphetamine; BACS-J=Brief Assessment of Cognition in Schizophrenia, Japanese Version; RAM=radial arm maze; NORM=novel object recognition test; Vas=visual analogue scale; PS=pain score; AIMS=involuntary movement scale; PANSS=positive and negative syndrome scale.

Therapeutic applications of YKS include the treatment of neurosis, insomnia, and night crying in children; some of these symptoms overlap with the nonmotor symptoms of PD. YKS may have therapeutic effects on PD, although many clinical and preclinical studies of YKS in other neurological disorders have also been done. Primarily, YKS has been shown to improve NPI scores, a measure of BPSD symptoms in patients with dementia and PD [2, 9–11, 14–17]. The neuropharmacological mechanisms underlying YKS's action include modulation of neurotransmitter systems, such those for serotonin, dopamine, glutamate, and GABA, as well as neuroprotection [24–30, 32–34, 36–39, 52, 53]. Apart from BPSD, limited data are available on the effects of YKS on the symptoms of PD, including autonomic dysfunction (mainly orthostatic hypotension, urogenital dysfunction, constipation, and hyperhidrosis) and sensory symptoms (most prominently, hyposmia). It remains necessary, however, to verify the complementary, therapeutic effects of YKS on the various symptoms of PD before it can be used with confidence to overcome the limitations of current PD therapeutics (Figure 1).

Conflicting reports on the effects of YKS have been made. For example, there is little evidence for YKS-mediated improvement in cognition among patients with PD in clinical trials, while positive results have been reported in preclinical studies. To resolve these differences, further research is needed to more appropriately select an optimal drug dosage, period of administration, and an evaluation index for use in clinical trials. Furthermore, preclinical models that more faithfully recapitulate the human PD condition are also needed. Prior to YKS being prescribed to patients with PD, its potential adverse effects must be considered and further research on them should be performed.

## Figures and Tables

**Table 1:** Clinical studies on the effects of YKS on PD-like symptoms in multiple neurological disorders.

**Symptom**	**Participants ** **(number)**	**Design**	**Intervention**	**Comparison**	**Outcome**		**Reference**
					**Primary**	**Secondary**	
Sleep	12 with AD and 1 with frontotemporal dementia (n= 13)	Open-label trial	YKS (5-7.5 g/day) for 8 weeks	None	Improved Sleep Disorder Inventory per NPI	Improved wake after sleep onset and total NPI scores; No change in MMSE	[5]
	5 with dementia	Open-label trial	YGS (2.5g) for 4 weeks	None	PSG; total sleep time, sleep efficiency, stage 2 sleep ↑ and the number of arousals and periodic limb movements ↓; Improved PSQI		[6]
	20 healthy adult males	Double-blind trial	YKSCH (7.5 g/day) for 3 days	Anchu-san	PSG; total sleep time, sleep efficiency, and stage 2 sleep ↑; Sleep latency and stage 3+4 sleep ↓		[7]
	3 with rapid eye movement sleep behavior disorder	Case report	YGS (7.5 g/day) + clonazepam (0.5 mg/day)	None	Suppression of RBD		[8]
			YGS (7.5 g/day) + clonazepam (0.25 mg/day)				
			YGS(2.5 g/day)				

	25 with PD	Open-label trial	YKS (7.5 g/day) for 12 weeks	None	NPI total score ↓; Improved subscale including hallucinations, anxiety, and apathy	No significant change in UPDRS part III and Hoehn–Yahr classification	[2]
	7 with PD 7 with PDD (n=14)	Open-label trial	YKS (7.5 g/day) for 4 weeks	None	NPI total score ↓; improved subscale including hallucinations MMSE improved slightly in PDD; No change in UPDRS III and ADL		[9]
	90 with dementia	Randomized rater-blinded trial	YKS (2.5-7.5 g/d) for 8 weeks	Risperidone or fluvoxamine	NPI-NH ↓	No change in MMSE, FIM, and drug-induced Extra-Pyramidal Symptoms Scale	[10]
	103 with dementia	Randomized cross-over trial	YKS (7.5 g) for 4 weeks	None	NPI total score ↓; improved subscale including delusions, hallucinations, agitation/aggression, depression, anxiety, and irritability/lability; No change in MMSE and ADL		[11]
	5 with dementia	Open-label trial	YGS (2.5 g) for 4 weeks	None	NPI-NH total score ↓; improved subscale including delusions, hallucinations, agitation/aggression, anxiety, and irritability/lability; No change in MMSE		[6]
Neuropsychiatry	52 with dementia	Randomized, observer-blind, controlled trial	YGS (7.5 g/day) for 4 weeks	Drug-free	NPI-NH total score ↓; improved subscale including hallucinations, agitation/aggression, irritability/lability and aberrant motor activity; Barthel index ↑; No change in MMSE		[12]
	145 with AD	Randomized double-blind placebo-controlled trial	YKS (7.5 g/day) for 4 weeks	Placebo-control	No significant difference in total NPI-Q scores	Improved NPI subscale including agitation/aggression and hallucinations; No change in MMSE	[13]
	61 with AD	Randomized, non-blinded, parallel-group comparison trial	YKS (7.5 g/day) + donepezil for 4 weeks	Active comparator	NPI total score ↓; improved subscale including agitation/aggression and irritability/lability; No significant difference in MMSE, DAD, ZBI, or SDS		[14]
	29 with AD	Open-label trial	YKS (7.5 g/day) for 4 weeks	None	NPI total score ↓	Clinically decreased subscale including hallucinations, agitation, anxiety, irritability, and abnormal behaviors; No significant difference in MMSE, DAD, ZBI, or SDS	[15]
	15 with AD	Randomized controlled trial	YKS(2.5 g) for 12 weeks + sulpiride (50 mg/day)	Control	NPI total score ↓; No change in MMSE and ADL		[16]
	13 with vascular dementia	Open-label clinical trial	YKS (7.5 g/day) for 4 weeks	None	NPI total score ↓; significantly improved subscale including agitation; No change in MMSE, ADL, DAD, or UPDRS		[17]
	40 with schizophrenia	Open-label clinical trial	YKS (2.5–7.5 g/day) for 4 weeks	None	Significantly improved BPRS, CGI-S, and PANSS; Slightly decreased Simpson-Angus scale, Barnes Akathisia scale, and AIM		[18]

Cognition	1 with schizophrenia	Case report	YKS (5.0 g/day) for 3 months	None	Markedly improved cognitive functions in daily life and BACS–J		[19]

Pain	10 with neuropathic pain	Open-label clinical trial		None	Markedly decreased VAS and PS		[20]

Tardive dyskinesia	22 with schizophrenia	Open-label trial	YGS (7.5 g/day) for 12 weeks	None	Significantly decreased AIMS total scores and all PANSS subscales; Substantial changes in CGI		[21]

NPI=neuropsychiatric inventory, MMSE=MINI-Mental State Examination, YGS=Yi-Gan San, PSG=polysomnography, ADL=the Barthel Index for activities of daily living, PDD=PD with dementia, NPI-NH=The Nursing Home version of Neuropsychiatric Inventory, PSQI=the Pittsburgh Sleep Quality Index, FIM=daily life function measured by Functional Independence Measure, DAD=Disability Assessment for Dementia, SDS=Self-rating Depression Scale, ZBI=Zarit Burden Interview, BPRS=psychiatric rating scale, CGI-S=clinical global impression scale-severity, PANSS=positive and negative syndrome scale, AIM=involuntary movement scale, BACS–J=Brief Assessment of Cognition in Schizophrenia, Japanese Version, VAS=visual analogue scale, and PS=pain score.

**Table 2:** Preclinical studies on the effects of YKS on PD-like symptoms in animal models of neurological disorders.

**Symptoms**	**Species**	**Inducer**	**Extracts/components**	**Dose/route/regimen**	**Major finding**	**Histological & biochemical evaluation**	**Reference**
Sleep	Male ddY mice (4 weeks old and 20–25g)	Social isolation for 9–12 weeks	YKS	300 mg/kg, p.o., 60 min prior to testing	Pentobarbital (50 mg/kg, i.p)-induced sleeping time ↑	Sleep inducing effect involving the GABA_A_ – benzodiazepine receptor complex, but not 5-HT_1A_ receptors	[22]
	Male ddY mice (4–6 weeks old and 19–32 g)		YKS	300 mg/kg, p.o.	Skin temperature ↓		[23]

Depression and anxiety	Male ICR mice (25–30 g)	Restraint stress for 14 days	YKS	1000 mg/kg, p.o.		Inhibition of decreased excitatory amino acid transporter 2 expression in the PFC and hippocampus	[24]

Anxiety	ICR male mice (6 weeks old)	Placed on elevated open-platform	YKSCH	800 mg/kg, p.o.	Duration of freezing in EPM ↓	Anxiolytic effects via a selective inhibitor of serotonin reuptake	[25]
	F344/N aged rats (24 months old)	Aging	YKS	3% (w/w) food pellets for 3 months	Time and frequency in the open arm of the EPM ↑; the number of excrements during locomotor activity measurement ↓	Improved concentrations of serotonin and dopamine in the PFC using microdialysis and NH3 in plasma	[26]
	Male Wistar/ST rats (10-13 weeks old)	Electric foot shock (aversive stressor)	YKS	1000 mg/kg, p.o., for 14 or 16 day	Freezing behavior ↓ in the CFC test; time spent in open arms ↑ in EPM test; unchanged locomotion	Anxiolytic effects via 5-HT_1A_ receptors in memory-dependent fear induced by aversive stress; plasma corticosterone (-) after CFC stress in rats that had experienced footshock stress	[27]
	Male ddY mice (4 weeks old)	Social isolation stress for 6 weeks	YKS	1% and 3% (w/w) food pellets for 6 weeks	Rearing behavior ↓	Anxiolytic effect mediated with 5-HT_1A_ receptor	[28]
	Male Wistar rats (250–300 g)	Cerebrovascular ischemia by the four-vessel occlusion method	YKS	100, 300, or 1000 mg/kg, p.o., for 14 days	The time in the dark box in the light/dark test and in the enclosed arm in EPM ↓; the proportion of counts in the outer area in OF test ↓; unchanged locomotion in the open-field	Antagonistic effect on wet-dog shakes induced by 5-HT_2A_agonist, DOI (5 mg/kg)	[29]
	Male ICR mice	CFC consisting of inescapable foot-shocks	Co-treatment SSRI (fluvoxamine) +YKS	Fluvoxamine (5–20 mg/kg, i.p.) and YKS (0.3 and 1 g/kg, p.o.) for 6 days	Freezing behavior characterized as anxiety behavior ↓ in CFC	Decreased 5-HT_2A_, but not 5-HT_1A_ receptor expression in the PFC	[30]
	F344/N male rats (10-13 weeks old)	Restraint stress for 1 h	YKS	100 or 300 mg/kg, p.o., for 1 h (single) or for 2 weeks (repeated)	Anxiety-related behavior in repeated administration ↓; unchanged locomotion by OF and EPM test	Decreased stress-induced c-Fos expression in the medial PFC and the basolateral and medial amygdaloid nuclei; serum corticosterone levels (-)	[31]

Hallucination	Male ddY mice (4 weeks old)	Isolation stress for 6 weeks; DOI, 2.5 mg/kg (i.p.)	YKS	1% and 3% (w/w) food pellets for 6 weeks or oral	DOI-induced head-twitch response ↓	Down regulation of 5-HT_2A_ receptor density in the PFC	[32]
	Male ddY mice ( 4 weeks old and 20–25 g)	DOI, 5 mg/kg, i.p.	YKS	300 mg/kg, p.o., for 14 days repeated treatment	DOI-induced head twitch response ↓	Decreased expression of 5-HT_2A_ receptors in the prefrontal cortex	[33]

Aggressiveness	Male Wistar rats (7 weeks old)	Social isolation for 11-13 weeks	YKS	100, 300 mg/kg, p.o.	Aggressive behavior ↓ using aggression test		[34]
	Male ddY mice (5 weeks old)	Methamphetamine			Methamphetamine-induced hyperlocomotion ↓	Inhibition of hyperlocomotion via dopaminergic signaling	
	Male ddY mice (6 weeks old)	Injection of A*β* into the lateral ventricle of the brain	YKS	0.5 or 1.0 g/kg for 3 weeks	Aggressive behaviors including tail rattling, chase, and attack ↓		[35]
	Male Wistar rats (6 weeks old)	Injection of L-Glutamic acid into the right NBM	YKSCH	1.0 g/10 ml/g for 7 days	Aggressive behavior in resident-intruder tests ↓	Mediated by 5-HT_1A_ receptor stimulation	[36]
	Male Wistar rats (7 weeks old)	Injection of PCA	YKS	0.5 or 1.0 g/kg single or chronic (2 weeks) after PCA injection	Aggressive behavior ↓	Agonistic effect on 5-HT_1A_ receptors as buspirone (5-HT_1A_ agonist) and chronic treatment with YKS inhibited aggressive behavior in PCA-injected rats	[37]
	Male ddY mice (3 weeks old)	Isolated zinc-deficient diet for 2 weeks	YKS	Drinking water containing YKS 300 mg/kg	Aggressive behavior in resident-intruder test ↓	Suppression of glutamatergic neuron activity; MK-801 (NMDA receptor antagonist) attenuated aggressive behavior in zinc-deficient mice	[38]
	Male ddY mice (3 weeks old)	Zinc-deficient diet for 2 weeks	YKS	Drinking water containing YKS 300 mg/kg	Aggressive behavior in resident-intruder test ↓	Lower serum glucocorticoid levels and ameliorated increased glutamate and GABA concentrations in brains of zinc-deficient mice	[39]
	Male ddY mice (3 weeks old) and male Wistar rats (3 weeks old)	Zinc-deficient diet for 2 weeks	YKSCH	Drinking water containing YKSCH 300 mg/kg for 2 weeks	Aggressive behavior in resident-intruder test ↓	Attenuation of excess exocytosis at mossy fiber boutons induced with 60 mM KCl in hippocampus and intracellular Ca^2+^ level by GA (100–500 *μ*M) or GM (100 *μ*M)	[40]

Cognition	Tg2576 mouse (5-15 months old)	AD	YKS	Powdered diets containing 0.5 and 1.0% YKS for 10 months	Learning disturbance in the Morris water-maze test ↓; time spent in the open arms in EPM test ↑; hyperactivity in OF test ↓	No inhibition of histological deposition or amounts of A*β* observed in Tg(+) mice	[41]
	Male Wistar rats (300–350 g)	Repeated cerebral ischemia	YKS and Angelica root	1000 mg/kg, p.o. for 7 days	Correct choice in 8 RAM task ↑	Upregulated the release of ACh; prevented 4-VO-induced hippocampal apoptosis	[42]
	Male Wistar rats (230–270 g)	Four-vessel transient cerebral ischemia and Injection of A*β* into intracerebroventricularly	YKS	100, 300, or 1000 mg/kg, p.o. for 2 weeks	Correct choices ↑ and error choices ↓ in the 8 RAM task	Increase of high K^+^-evoked potentiation of ACh release and expression of dynamin 1 in the hippocampus and leading to improved synaptic function	[43]
	OBX male ddY mice (9 weeks old)	AD	YKS	375–750 mg/kg, p.o.	Memory deficits in modified Y-maze test and novel object recognition test ↓; long term memory impairment in fear conditioning test (-)	Reversed OBX-induced down-regulation of ChAT and muscarinic M1 receptor expression without affecting muscarinic M3 receptor expression or AChE activity	[44]
	Naive male F344/N rats (24 months old)	Aging	YKS	3% (w/w) food pellets for 3 months	Accuracy in the delayed alternation ↑ (working memory); accuracy in reversal discrimination tasks ↑ (reversal learning)	Increase of dopaminergic signaling through D1 receptors in the PFC as the effect of YKS was reduced by intracranial infusion of a dopamine D1 receptor antagonist	[45]
	Male Wistar rats (3 weeks old and 35-45g)	Thiamine-deficient diet	YKS	Drinking water containing YKS 0.5, 1.0 g/kg	Memory disturbance in step-through passive-avoidance test ↓	Inhibition of neuronal and astroglial cell degeneration in the brainstem, hippocampus, and cortex; inhibition of extracellular glutamate rise in the ventral posterior medial thalamus	[46]
	Male homozygous (j/j) Gunn rats (7 weeks old)	Gunn rat, animal model of schizophrenia	YKS	Drinking water containing YKS 1 g/kg	Location index in object-location test ↑	Suppression of microglial activation and promotion of neurogenesis in the hippocampus	[47]

Motor	Male Mongolian gerbils (60–80 g)	Cerebral ischemia by occlusion of bilateral common carotid arteries for 5 min	YKS	300 mg/kg, p. o. for 30 days	Ischemia-induced locomotor hyperactivity ↓; errors recorded in the 8-RAM task ↓	Decreased number of activated microglia in the CA1 based on Iba1 immunohistochemistry; amelioration of ischemia-induced oxidative DNA damage via 8-OHdG assay	[48]

Pain	Male SD rats (250-300g)	Chronic constriction injury	YKS	0.3, 1.0 g/kg, p.o.	Mechanical allodynia in the von Frey filament test ↓; cold allodynia in acetone test ↓	Inhibition of cerebrospinal fluid dialysate levels of glutamate; DL-threo-beta-hydroxyaspartate and dihydrokainate as glutamate transporter inhibitor reduced the antiallodynic effects of YKS	[49]
	Male ICR mice (5 weeks old)	Partial sciatic nerve ligation	YKS	1 g/kg, p.o.	Mechanical allodynia in the von Frey filaments test ↓	Inhibition of *IL-6* mRNA expression in astrocytes and/or microglia in the spinal cord	[50]

Tardive dyskinesia	Male Wistar rats (5 weeks old)	Injection of haloperidol decanoate-into thigh muscle	YKS	0.1, 0.5 g/kg, p. o. for 3 weeks	Vacuous chewing movement ↓	Inhibition of increased striatal glutamate and *GLT-1* mRNA levels in haloperidol-treated rats	[51]

DOI=1-(2,5-dimethoxy-4-iodophenyl)-2-aminopropane, CFC=contextual fear conditioning, EPM=elevated plus-maze, OF=open-field, PFC=prefrontal cortex, Ach=acetylcholine, RI=repeated ischemia, ChAT=choline acetyltransferase, AchE=acetylcholinesterase, RAM=radial arm maze, YKSCH=Yokukansankachimpihange, PCA=para-chloroamphetamine, AD=Alzheimer's disease, A*β*=amyloid *β*, GA=18*β*-glycyrrhetinic acid, GM=geissoschizine methyl ether, and 4-Vo=4-vessel occlusion.

**Table 3:** Neuroprotective effect of YKS on various *in vitro* systems.

**Model**	**Cell type**	**Inducer**	**Extracts/components**	**Dose/route/regimen**	**Major finding**	**Histological & biochemical evaluation**	**Reference**
Neuroprotection	Hippocampal neurons	Corticosterone (CORT)	YKS	100-1000 *μ*g/mL, p.o.; five times every 12 h before cocaine treatment	Cell survival rates measured by the WST-8 and LDH assays ↑		[52]
	Rat cortical neurons	20 *μ*M A*β*-induced cytotoxicity	YGS	10^−5^ g/L (w/v) for 24 h before exposure to 20 *μ*M A*β*	The surviving cortical neurons determined by MTT and LDH assays ↑		[53]

Oxidative stress	PC12 cells	Glutamate (1–17.5 mM)-induced cytotoxicity	YKS	125-500 *μ*g /mL	Protection against PC12 cell death determined by MTT assay	Reduction of intracellular GSH level ↓	[54]
	PC12 cells	Hydrogen peroxide (H_2_O_2_)-induced apoptosis	YGS40	100 *μ*mol/L of H_2_O_2_ for 12 h	Cytotoxicity determined by MTT and LDH assays ↓	Annexin V-positive ↓; caspase-3 activity ↓; Bax ↓; Bcl-2 ↑; MMP loss ↓; activity of SOD ↑; MDA generation ↓	[55]

Neurotransmission	Chinese hamster ovary cells	Competitive binding for 5-HT receptors on CHO (CHO-h5-HT1A) or CHO-K1 (CHO-K1-h5-HT2A) cells	YKS	6.25-400 *μ*g /mL	Partial agonistic effect on 5-HT_1A_ receptors		[56]

LDH=lactate dehydrogenase, MMP=mitochondrial membrane potential, SOD=superoxide dismutase, and MDA=malondialdehyde.
